# Exploring adverse drug events at the class level

**DOI:** 10.1186/s13326-015-0017-1

**Published:** 2015-05-01

**Authors:** Rainer Winnenburg, Alfred Sorbello, Olivier Bodenreider

**Affiliations:** Center for Biomedical Informatics Research, Stanford University, Stanford, CA USA; Center for Drug Evaluation and Research, US Food and Drug Administration, Silver Spring, MD USA; Lister Hill National Center for Biomedical Communications, National Library of Medicine, National Institutes of Health, Bethesda, MD USA

**Keywords:** Adverse drug events, Drug classes, Anatomical Therapeutic Chemical (ATC) drug classification system, Class effect, Heat maps, Pharmacovigilance

## Abstract

**Background:**

While the association between a drug and an adverse event (ADE) is generally detected at the level of individual drugs, ADEs are often discussed at the class level, i.e., at the level of pharmacologic classes (e.g., in drug labels). We propose two approaches, one visual and one computational, to exploring the contribution of individual drugs to the class signal.

**Methods:**

Having established a dataset of ADEs from MEDLINE, we aggregate drugs into ATC classes and ADEs into high-level MeSH terms. We compute statistical associations between drugs and ADEs at the drug level and at the class level. Finally, we visualize the signals at increasing levels of resolution using heat maps. We also automate the exploration of drug-ADE associations at the class level using clustering techniques.

**Results:**

Using our visual approach, we were able to uncover known associations, e.g., between *fluoroquinolones* and *tendon injuries*, and between *statins* and *rhabdomyolysis*. Using our computational approach, we systematically analyzed 488 associations between a drug class and an ADE.

**Conclusions:**

The findings gained from our exploratory techniques should be of interest to the curators of ADE repositories and drug safety professionals. Our approach can be applied to different drug-ADE datasets, using different drug classification systems and different signal detection algorithms.

**Electronic supplementary material:**

The online version of this article (doi:10.1186/s13326-015-0017-1) contains supplementary material, which is available to authorized users.

## Background

### Motivation

According to the Agency for Healthcare Research and Quality (AHRQ), adverse drug events (ADEs) “result in more than 770,000 injuries and deaths each year and cost up to $5.6 million per hospital” [1]. Drug safety is addressed through the drug development process, not only during clinical trials [2], but also through postmarketing surveillance, by analyzing spontaneous reports [3], observational data [4] and the biomedical literature [5].

While the association between a drug and an adverse event is generally detected at the level of individual drugs (e.g., between aspirin and Reye syndrome [6]), ADEs are often discussed at the level of pharmacologic classes. Examples include the ototoxicity of aminoglycosides [7], the association between statins and rhabdomyolysis [8], and between vaccines and Guillain-Barré syndrome [9]. These examples illustrate the need for investigating ADEs at the class level, i.e., after aggregating individual drugs into pharmacologic classes.

Some ADEs can be observed with every individual drug in a class. This is often the case when the ADE is related to the physiologic effect of the drug. For example, bleeding is a common effect of anticoagulants, such as vitamin K antagonists [10]. Conversely, some ADEs are associated with some class members, but not with all of them. For example, a recent review reports a differential risk of tendon injuries with various fluoroquinolones, the highest risk being with ofloxacin [11].

From an ontological perspective, it is interesting to explore whether the ADE is an inherent property of the class (inherited by every member of the class) or rather a property of some members only. In practice, when there is a high risk of an ADE for a class (i.e., a strong class-level signal), one may want to “drill down” and investigate the drug-level signal for each individual drug in the class to discover if the class-level signal results from uniformly high drug-level signals, or is rather driven by an intense signal for a small number of drugs, while the other drugs in the class would not exhibit a high risk for this ADE. The former reflects a “class property” inherited by each drug, whereas the latter reflects a “drug property”, i.e., a property for some of the drugs only.

The objective of this work is to explore the contribution of individual drugs to the class signal. More specifically, we propose two approaches, one visual and one computational, to identifying “class effects”, i.e., cases when all drugs in a class have the same ADE (as opposed to cases where the class signal is driven by only a few drugs from the class).

### Drug and ADE terminologies

The following sections detail the characteristics of the resources used in this research. We use MeSH for aggregating ADEs and ATC for drug classification purposes. We also use RxNorm to harmonize drugs between MeSH and ATC.

#### MeSH

The MeSH thesaurus is the controlled vocabulary used to index documents included in the MEDLINE database [12]. It contains over 27,000 descriptors (main headings) organized in sixteen hierarchical tree structures. Each tree contains up to eleven levels denoting aboutness relationships between the terms. For example, the term *Rhabdomyolysis* is classified under *Muscular Diseases* in the *Diseases* tree. Version 2014 of MeSH is used in this study.

#### ATC

The Anatomical Therapeutic Chemical (ATC) classification [13], a system developed by the World Health Organization (WHO) Collaborating Centre for Drug Statistics Methodology, is recommended for worldwide use to compile drug utilization statistics. The system includes drug classifications at 5 levels; anatomical, therapeutic, pharmacological, chemical and drugs or ingredients. For example, the 4th-level ATC class *Vitamin K antagonists* (B01AA) has the following 5th-level drugs as members: *acenocoumarol*, *dicumarol*, *fluindione*, *phenindione*, *phenprocoumon*, *tioclomarol* and *warfarin*. The 2014 edition of ATC used in this study contains 4,580 5th-level ATC drugs and 1,256 drug classes.

#### RxNorm

RxNorm is a standardized nomenclature for medications produced and maintained by the U.S. National Library of Medicine (NLM) [14]. Both ATC and MeSH are integrated in RxNorm, making it possible for us to use RxNorm to link MeSH drugs to their classes in ATC. Moreover, RxNorm provides a rich network of relations among various types of drug entities, making it possible to normalize the various salts and esters of a drug (“precise ingredients”) to their base form (“ingredient”). The April 2014 version of RxNorm is used in this study and was accessed through the RxNorm API [15].

### Related work

#### ADE extraction and prediction

There is a large body of research on the extraction of drug ADE associations from various sources (e.g., [3-5,16]), in which terminologies are usually leveraged for the normalization of drugs (e.g., to RxNorm and ATC) and adverse reactions, for example to the Common Terminology Criteria for Adverse Events (CTCAE) and the Medical Dictionary for Regulatory Activities (MedDRA). Researchers have also created repositories of ADEs, such as ADEpedia [17] and used network analysis to analyze and predict drug-ADE associations [18]. In our effort to explore the ADEs at the class level, we use an existing dataset of drug-ADE pairs obtained from prior work on extracting drug-ADE pairs from MEDLINE indexing.

#### Research on class effect

Many researchers have investigated whether a given ADE was specific to a drug or common to all drugs in the corresponding class. Examples of such investigations include the exploration of antiepileptic-induced suicidality [19], association between anti-VEGF agents and dysthyroidism [20] or avascular necrosis of the femoral head [21], association between dipeptidyl-peptidase-4 inhibitors and heart failure [22] or angioedema [23], and atypical antipsychotic-induced somnambulism [24]. A search for “class effect” in the titles of PubMed articles retrieves over one hundred citations. Such efforts, however, generally investigate one specific drug class and one specific ADE. In contrast, we propose a method for assessing the class effect over a wide range of drug classes and ADEs.

### Specific contribution

The specific contribution of our work is to combine existing drug safety signal detection and visualization techniques, and to leverage drug terminologies for exploring adverse drug events at the class level. We extend the visual exploration with an automated computational approach to identifying class effects, allowing their systematic detection from any dataset of drug-ADE associations.

## Methods

Our approach to exploring ADEs at the class level can be summarized as follows. We first establish a dataset of ADEs by extracting drug-ADE pairs from MEDLINE. Then we aggregate drugs into ATC classes and ADEs into high-level MeSH terms. We compute the association between drugs and ADEs at the drug level and at the class level. In our visual approach, we use heat maps to visualize the signal at increasing levels of resolution to distinguish between drug-level and class-level ADEs. In our computational approach, we achieve the same result by leveraging clustering techniques. While the visual approach requires manual selection of the classes and ADEs of interest, the computational approach is completely automated and can be applied over a wide range of drug classes and ADEs.

### Extracting drug – adverse event pairs from the literature

Our dataset consists of pairs of drugs and ADEs extracted from the MEDLINE database, using an approach similar to [5]. We use combinations of MeSH descriptors (and supplementary concepts) and qualifiers to identify, on the one hand, drugs involved in ADEs (e.g., *ofloxacin*/*adverse effects*) and, on the other, manifestations reflecting an ADE (e.g., *tendinopathy*/*chemically induced*). We improved upon [5] by also taking into account those MeSH descriptors inherently indicative of adverse events (e.g., *Drug-induced liver injury*). We collected the resulting list of drug-manifestation pairs for each ADE (e.g., *ofloxacin*-*tendinopathy*).

### Linking MEDLINE drugs to ATC classes

We map all MeSH drugs extracted from MEDLINE to our target terminology, ATC, for aggregation purposes, using RxNorm.

#### Mapping MeSH drugs to ATC drugs through RxNorm ingredients

Both ATC and MeSH are integrated in RxNorm. For example, the RxNorm drug *rosuvastatin* (301542) is linked to both the MeSH drug *rosuvastatin* (C422923) and the 5th-level ATC drug *rosuvastatin* (C10AA07). Individual drugs in MeSH correspond to ingredients (IN) and precise ingredients (PIN) in RxNorm. We normalize the drugs by mapping PINs to their corresponding INs. For example, RxNorm explicitly asserts that *valproic acid* is the “precise ingredient” of the ingredient *valproate*.

Of note, a given drug can be represented multiple times in ATC. Typically, topical drugs and systemic drugs have different ATC codes for the same active moiety. For example, the anti-infective *ofloxacin* has two codes in ATC, depending on whether it is classified as an antibacterial drug for systemic use (J01MA01) or as an ophthalmological drug (S01AE01). However, we consider unique drugs, not multiple codes, when we associate drugs with their ADEs. We only use the codes to link drugs to their classes. The individual MeSH drugs extracted from MEDLINE and which map to ATC constitute the set of eligible drugs for this study.

#### Establishing drug class membership

In ATC, the 5th-level drugs are linked to one or more 4th-level classes. For example, *ofloxacin* is a member of the two *Fluoroquinolones* drug classes (J01MA and S01AE). For the purpose of comparing class-level ADEs to drug-level ADE, we require that the classes contain a sufficient number of members. In practice, we exclude all drug classes with fewer than 4 drug members in our set of drugs. In this proof-of-concept investigation, this threshold was selected as a trade-off between retaining a sufficient number of classes and getting a meaningful interpretation of the characteristics of the drugs in these classes.

### Aggregating adverse event terms in MeSH

ADEs can be expressed at different levels of granularity. The MeSH hierarchy has multiple levels, enabling MEDLINE indexers to capture information at the appropriate level of granularity. However, for analytical purposes, it is useful to aggregate detailed ADEs into coarser ADE classes, similarly to what we do for the drugs. We use descriptors at the second level of the MeSH hierarchy for aggregation purposes. For example, we would aggregate *Tendinopathy* (tree number C05.651.869) and *Rhabdomyolysis* (C05.651.807) to the second-level descriptor *Muscular Diseases* (C05.651).

### Computing adverse event signals at the drug level

In pharmacovigilance, safety signal detection consists in the identification of an association between a drug and an adverse event (AE). In this study, we use the traditional proportional reporting ratio (PRR) [25] in computing statistical associations for unique drug- and drug class-AE pairs. PRR is a simple disproportionality method for signal detection that is easy to compute and sufficient in the context of this study. Based on the frequencies shown in Table 1, the PRR is defined as follows:

**Table 1:** **Example of contingency table representing drug-ADE associations in MEDLINE**

	***With this ADE***	***Without this ADE***
Articles mentioning this drug	a	b
Articles not mentioning this drug	c	d

1 $$ PRR=\left(a/\left(a+b\right)\right)/\left(c/\left(c+d\right)\right) $$

We calculate signals for all possible combinations of drugs and ADEs that co-occur in at least one MEDLINE article. We apply the usual zero-cell correction to tables where b or c is equal to 0 (by adding 0.5 to each count in the 2 × 2 table). For all pairs that do not co-occur in the literature, we set the PRR to a neutral value of 1.

### Computing adverse event signals at the class level

At the class level, we compute the signal using a similar approach. For drug classes, we count articles mentioning any drug from this drug class (a and b) and articles mentioning any other drug (c and d). For ADE classes, we count articles with any ADE from this ADE class (a and c) and articles with any other ADE (b and d).

### Exploring ADE signals at different levels

We want to determine whether the class signal is driven by the strong signal of only a few drugs or is distributed among all drugs from that class. To this end, we visually explore the signal at different levels of granularity, from drug class-ADE class, to individual drug-ADE class, to individual drug-individual ADE. Visual patterns reflect the contribution of the drug signal to the class signal. We draw on the techniques popularized by gene expression data studies, combining clustering and “heat map” visualization [26], for exploring the relations between drugs and ADEs. We rely on the R statistical software package (version 3.1.2) for implementation. More specifically, we use *hclust* for clustering (using complete linkage and Euclidean distance) and *heatmap* for visualization.

#### Drug class-ADE class signal

We start by plotting all ATC4 drug classes against all ADE classes, using the drug class signal. To reduce the amplitude of the PRR signal, we plot the log_n_ transform of the PRR for all eligible class pairs. We perform hierarchical clustering on both drug classes and ADE classes to group pairs of drug classes and ADE classes with similar signals. On the resulting heat map, strong signals will appear in white and yellow, while weak signals will be displayed in red.

#### Drug-ADE class signal

**Figure 1:**
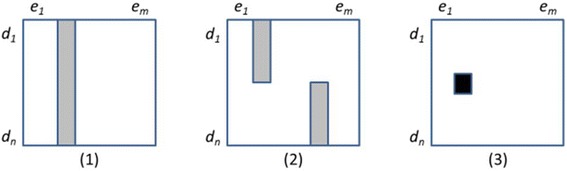
Patterns of associations between members of drug class *C*_*D*_ *(d*_*1*_ *,..,d*_*n*_ *)* and the manifestation of an adverse event class *C*_*E*_ *(e*_*1*_ *,..,e*_*m*_ *).*

While a low-resolution map is sufficient to identify strong class signals and the corresponding broad ADE classes, a higher resolution is required to investigate the distribution of the class signal among the individual drugs members. Starting from the strongest signals observed in the previous step for a given drug class (e.g., PRR above 10), we plot the signal for each drug in the class. Here again, we perform hierarchical clustering of both individual drugs and ADE classes (based on the drug-level PRR, as opposed to the class-level PRR used in the previous step). This heat map exhibits the distribution of the class signal among the individual drug members. In some cases, we see the emergence of characteristic patterns illustrated in Figure 1:

1. A solid column (vertical bar) with medium intensity (bright orange/ yellow) reflects an ADE (class) that is equally distributed among all members of the class, corresponding to a “class property”.
2. Several incomplete, non-overlapping vertical bars in different columns, with medium intensity, reflect ADEs (ADE classes) associated with subsets of the class members, but not all members. This pattern corresponds to the properties of sets of individual drugs, rather than the property of the class itself.
3. Isolated spots or small islands of high intensity reflect associations between one drug (or few drugs) from the class and an ADE (class), corresponding to individual drug properties.

#### Drug-ADE signal

Finally, to assess individual ADEs, we plot the drug-level signal for each ADE in the ADE classes present at the previous step. As before, we perform hierarchical clustering on both drugs and ADEs (based on the drug-level PRR). This heat map exhibits the distribution of the ADE class signal among the individual ADEs. Patterns similar to those described above can also be observed.

### Automating the detection of class effects

While the visual approach provides an intuitive exploration of the ADEs within a drug class, its manual nature restricts its large applicability. Here we propose an automated approach to identifying class effects in the same dataset.

#### Intuition

In case of a class effect, the PRRs are expected to be homogeneous among all drug members in a class for a given ADE, and we should not be able to identify distinct subgroups among them. Conversely, if we can identify subgroups among the drugs, it means that the class signal is driven by some drugs more than others, which is not characteristic of a class effect.

#### Implementation

For a given drug class and ADE pair, we have computed the class-level signal (as described in section 2.5) and the drug-level signal for each drug in the class (as described in section 2.4). Only classes with at least four drug members are considered. Because PRRs are proportions, we use their log_n_-transformed value to approach a normal distribution.

To examine the distribution of the PRRs for individual drugs in the class, we use k-means clustering with Euclidean distance to identify two clusters (k = 2) among the (log_n_-transformed) PRRs. We then compare the means between the two clusters using Welch's t-test, which accommodates unequal variances in samples. Of note, in some cases, when the PRRs for all drugs in a class are very similar, k-means clustering only produces a single cluster. In this case, we assume that this cluster is homogeneous by design. When we obtain only one cluster or when the hypothesis of a difference between the means of the two clusters is rejected (p-value > 0.05), we conclude to a class effect.

For example, the 4th-level ATC class *selective serotonin reuptake inhibitors* (N06AB) has a (log_n_-transformed) PRR of 4.30 for the ADE *sexual dysfunctions*. We partition the PRRs for the individual drugs into two clusters: {*fluoxetine* (4.25), *fluvoxamine* (3.85)} and {*sertraline* (3.68), *citalopram* (3.57), *paroxetine* (3.77), *escitalopram* (3.57)}. There is no significant difference between the means of the two clusters (p-value 0.28). Thus we conclude that all the individual drugs contribute to the signal for the drug class, which is the characteristic of a class effect.

## Results

### Drug-ADE dataset

**Figure 2:**
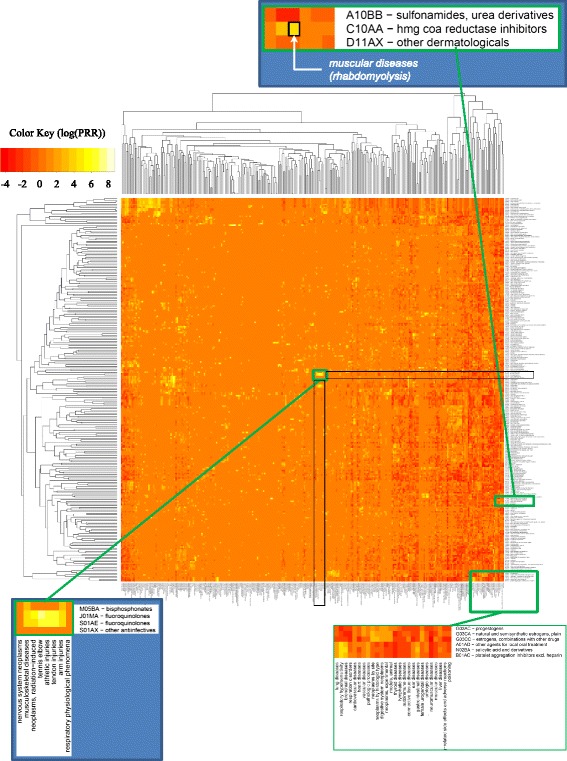
Heat map of drug classes and ADE classes (based on the class signals).

We collected 189,800 MEDLINE articles, from which we extracted 371,417 drug-ADE pairs. The 244,692 MeSH drug instances mapped to 1,966 distinct 5th-level ATC drugs, and were aggregated into 598 4th-level ATC classes, of which 261 had at least four drugs. The 282,691 adverse event instances (3,043 distinct MeSH terms) were aggregated into 314 2nd-level descriptors in MeSH. The coarse matrix (Figure 2) reflects the association between each of the 261 drug classes of interest and the 314 ADE classes. The dataset used for our computational approach includes all the 3,043 individual ADEs for each of the 261 drug classes under investigation (794,223 pairs).

### Visual approach

#### Drug class-ADE class signal

Figure 2 represents the heat map of 261 drug classes and 314 ADE classes, with drug classes in rows and ADE classes in columns. Because of the large number of classes, the labels are not legible at this resolution. (A high-resolution version of the heat maps is available as Additional file 1). However, bright yellow spots or islands are clearly visible. For example, the yellow rectangle right at the center corresponds to the association between *fluoroquinolones* and various kinds of tendon injuries. Isolated bright spots are equally interesting. For example, the strong signal between *statins* and *muscular diseases* is represented by a single bright spot.

#### Drug-ADE class signal

**Figure 3:**
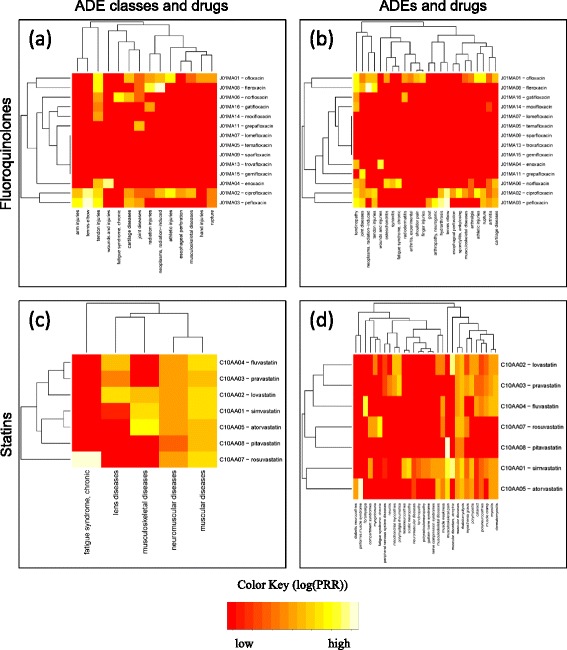
Detailed heap maps for individual drug classes (based on the individual drug signals); a) Fluoroquinolones, ADE classes and drugs; b) Fluoroquinolones, ADEs and drugs; c) Statins, ADE classes and drugs; d) Statins, ADEs and drugs.

The left part of Figure 3 shows examples of interesting patterns. There is a solid bar for all members of the *statins* class and the ADE class *muscular diseases*. And there is an incomplete column involving 8 of the 14 members of the *fluoroquinolones* class for the ADE class *tendon injuries*. Isolated spots are also visible, for example, between *rosuvastatin* and *chronic fatigue syndrome*, and between *fleroxacin* and *radiation injuries* and *radiation-induced neoplasms*.

#### Drug-ADE signal

The right part of Figure 3 also shows examples of interesting patterns, with higher resolution than before. For example, the solid bar between the *statins* class and the ADE class *muscular diseases*, visible on the left, is conserved, but we can now see that its signal is driven by the specific ADE *rhabdomyolysis*.

### Computational approach

Of the 794,223 pairs of (drug class, ADE), the large majority correspond to cases where at least one of the drugs in the class has no reported association with the ADE in the pair. In the visual approach, we assigned such combinations a neutral PRR of 1 for display purposes, resulting in many “red” areas on the heat map. In the computational approach, however, we ignored such cases, because we cannot distinguish between absence of evidence and evidence of absence for the drug-ADE association. As a consequence, only 488 drug class-ADE pairs could be explored for class effect. The class PRRs for these pairs ranged from 0.11 to 373.97 (before log_n_ transformation), with 134 pairs having a PRR above 10 and 214 pairs having a PRR above 5.

The clustering process yielded two clusters in 457 cases (93%) and a single cluster in 31 cases (7%). When two clusters were identified, the difference between their means was not significant in 337 (74%) and significant in 120 (26%) of the 457 cases. Of note, a significant difference between the clusters does not necessarily rule out the possibility of a class effect, because the average PRRs may be high in both clusters.

Examples of pairs with a single cluster include (*corticosteroids*, *femur head necrosis*) and (*fibrates*, *muscular diseases*). Examples of pairs with two clusters between which no difference could be found include (*tetracycline and derivatives*, *tooth discoloration*), (*statins*, *rhabdomyolysis*) and (*selective serotonin reuptake inhibitors*, *sexual dysfunctions, psychological*). In many of the pairs with two significantly different clusters, the PRRs were high in both clusters, suggesting a class effect despite the presence of two distinct clusters. For example, in the pair (*other aminoglycosides*, *labyrinth diseases*) the average PRR is 57 in the first cluster (7 drugs) and over 350 in the second cluster (2 drugs). While drugs from the second cluster (*arbekacin* and *dibekacin*) show a higher risk of ototoxicity, the risk for the drugs from the first cluster seems high enough (PRR = 57) for labeling ototoxicity a class effect. In contrast, there are pairs with two significantly different clusters where the PRRs are high in one cluster and low in the other. For example, in the pair (*selective serotonin reuptake inhibitors*, *long QT syndrome*), only the drugs *citalopram* and *escitalopram* exhibit a high PRR (about 20), while other drugs from this class have low PRRs (e.g., *sertraline* and *paroxetine* have PRRs between 1 and 2).

## Discussion

### Findings

#### Visual approach

Using our visual approach to exploring ADEs at the class level, we were able to uncover known associations, e.g., between *fluoroquinolones* and *tendon injuries*, and between *statins* and *rhabdomyolysis*. More specifically, exploring the signal at increasingly higher levels of resolution revealed a difference between *fluoroquinolones* and *statins*. Although both drug classes exhibit a strong class-level signal for their respective ADEs, only 8 of the 14 individual *fluoroquinolones* showed an association with *tendon injuries*, while all *statins* were associated with *rhabdomyolysis*. This difference illustrates the distinction between a class effect (*statins*), i.e., inherited by all members, and the property of a subset of the class members.

#### Computational approach

The computational approached proposed here automates the interactive strategy for exploring the class signal introduced with the visual approach. The patterns detected on the heat map (Figure 1) correspond to cases where all drugs from the class have roughly similar PRRs (solid bar), or where groups of drug with different PRR levels can be found (incomplete bar or isolated spot). Translated into clusters for automated processing, the solid bar corresponds to a single cluster or two clusters with similar PRR levels (no significant difference between the clusters), while the incomplete bar corresponds to two distinct clusters with significant difference between their average PRR levels. For example, for the pair (*statins*, *rhabdomyolysis*), we found two clusters with no significant difference. In contrast, the pair (*fluoroquinolones*, *tendon injuries*) was excluded from automatic processing, because association with *tendon injuries* had been reported for only four drugs (*ciprofloxacin*, *fleroxacin*, *pefloxacin* and *ofloxacin*), while no information was available for the other ten fluoroquinolones in this class (e.g., *trovafloxacin*). In this case, expertise is required to distinguish between less toxic drugs and drugs recently marketed for which no ADEs have been reported as of yet. For this reason, a proper determination of class effect could be suggested for only 488 pairs based on the dataset we exploited.

### Applications

The findings gained from our exploratory techniques should be of interest to the curators of ADE repositories and drug safety professionals. One drug safety issue has to do with the information found in drug labels, where ADEs can be labeled in reference to a specific drug or to an entire class of drugs. For example, the drug label for *citalopram* includes a warning for *QT prolongation* (not found in other SSRIs, such as *sertraline*). In contrast, the label for *minocycline* refers to an ADE for its class: “THE USE OF DRUGS OF THE TETRACYCLINE CLASS DURING TOOTH DEVELOPMENT […] MAY CAUSE PERMANENT DISCOLORATION OF THE TEETH”. To make this determination, drug safety officers must be able to access not only safety information for a given drug, but also safety information for the other members of its class. The approaches we propose here support effective review of safety information in the context of drug classes.

To assess the relevance of our determination of a potential class effect with respect to information found in the FDA-approved structured package labels available as part of DailyMed [27], one of the authors (AS) with a drug safety background reviewed the top-20 pairs selected by our computational approach. These pairs are 20 of the 488 pairs with the highest class-level PRR (>40), for which 2 clusters had been identified, but no significant difference between the clusters had been found. These pairs included well-known class effects mentioned in drug labels, including (*tetracycline and derivatives*, *tooth discoloration*), (*statins*, *rhabdomyolysis*) and (*selective serotonin reuptake inhibitors*, *sexual dysfunctions, psychological*) and (*selective serotonin reuptake inhibitors*, *serotonin syndrome*). In five cases, the ADE is mentioned for all the drugs in the class, but the drug label does not make explicit reference to the class in the warning. In six other cases, it was not possible to verify the information because there was no label available for some of the drugs in the class (e.g., drugs not marketed in the U.S.). Finally, the remaining cases included false positives, where an ADE known to be associated with a given systemic drug was wrongly associated with topical forms of the drug (because our underlying dataset does not contain information about routes of administration).

Overall, these results suggest that, while potentially helpful to drug safety officers for exploring ADEs for drugs in the context of their classes, our approaches to identifying class effect should only be used to support determinations made by domain experts.

### Limitations and future work

A vast majority of the drug class-ADE pairs explored by our computational approach ended up not being amenable to class effect determination, because no ADE information was retrieved for at least one of the drugs in the class. Our class definitions are based on ATC and included drugs not marketed in the U.S., which made it difficult to compare this information with warnings contained in the drug labels from DailyMed. Restricting the definition of drug classes to U.S. marketed drugs would have led to a more meaningful comparison with DailyMed information. Moreover, having additional information about the drugs would allow us to distinguish between older drugs for which no ADEs have been mentioned (i.e., evidence of absence for the ADE) and drugs more recently marketed for which there has not been enough time for collecting safety information through case reports (i.e., absence of evidence for the ADE).

Also missing from our current approach is an assessment of the strength of evidence for the drug-ADE signal based on study design. For example, randomized clinical trials could be given preference over non-comparative observational studies and case reports [28] . However, because our dataset is extracted from the biomedical literature, we could easily provide supporting information, such the number of articles in which the ADE is reported for the drugs, as well as the publication type (e.g., case report vs. clinical trial).

We are aware that our dataset of drug-ADE pairs extracted from the biomedical literature is biased (e.g., towards case reports). However, our approach is agnostic to the source used to derive the signal. In future work, we are planning to apply it to the data from the FDA Adverse Event Reporting System (FAERS). We could also leverage natural language processing (NLP) techniques to extract ADE pairs from text. Advanced NLP techniques would be able to extract the polarity of ADEs (i.e., negated ADEs), helping to assess evidence of absence of ADEs.

The signal detection method used in this investigation is extremely simple and may not be as robust as disproportionality score algorithms developed more recently. For example, limitations inherent in the use of PRR include inability to account for temporal trends and confounding by age, sex, or concomitant drugs [29]. Here again, our approach is agnostic to the methods used for signal detection and could easily be adapted to more sophisticated scores.

Finally, while aggregation plays a central role in our approach, ATC and MeSH are not the only terminologies that can support aggregation. For example, the Established Pharmacologic Classes distributed by FDA together with the Structured Product Labels may offer an alternative drug classification system. Our method for aggregating ADEs in MeSH was limited to one level across all subdomains and would benefit from refinement. Also, terminologies such as MedDRA offer not only an alternative, but groupings of ADEs across hierarchical structures.

## Conclusions

We presented two complementary approaches to exploring the contribution of individual drugs to the class signal for ADEs. The visual approach supports the interactive exploration of the class signal at increasing levels of resolution. We showed that specific visual patterns in heat maps are associated with class effects. Additionally, we presented a computational approach, complementary to the visual approach, meant to assess the class effect over a wide range of drug classes and ADEs systematically and automatically. In both cases, we were able to find support for multiple known class effects. Some of our findings were difficult to corroborate against drug labels of DailyMed for a variety of reasons. Our approach can be applied to other drug-ADE datasets, using various drug classification systems and signal detection algorithms. The findings gained from our exploratory techniques should be of interest to the curators of ADE repositories and drug safety professionals.

## Additional file

Additional file 1:
**High-resolution heat maps of drugs and ADEs at different levels of granularity.**
