# Supplementary material for: Exploring adverse drug events at the class level
Source: J Biomed Semantics. 2015 May 1;6:18. doi: 10.1186/s13326-015-0017-1 (PMC4416343; doi:10.1186/s13326-015-0017-1)
Supplement: Additional file 1: — High-resolution heat maps of drugs and ADEs at different levels of granularity. [file 13326_2015_17_MOESM1_ESM.pdf]

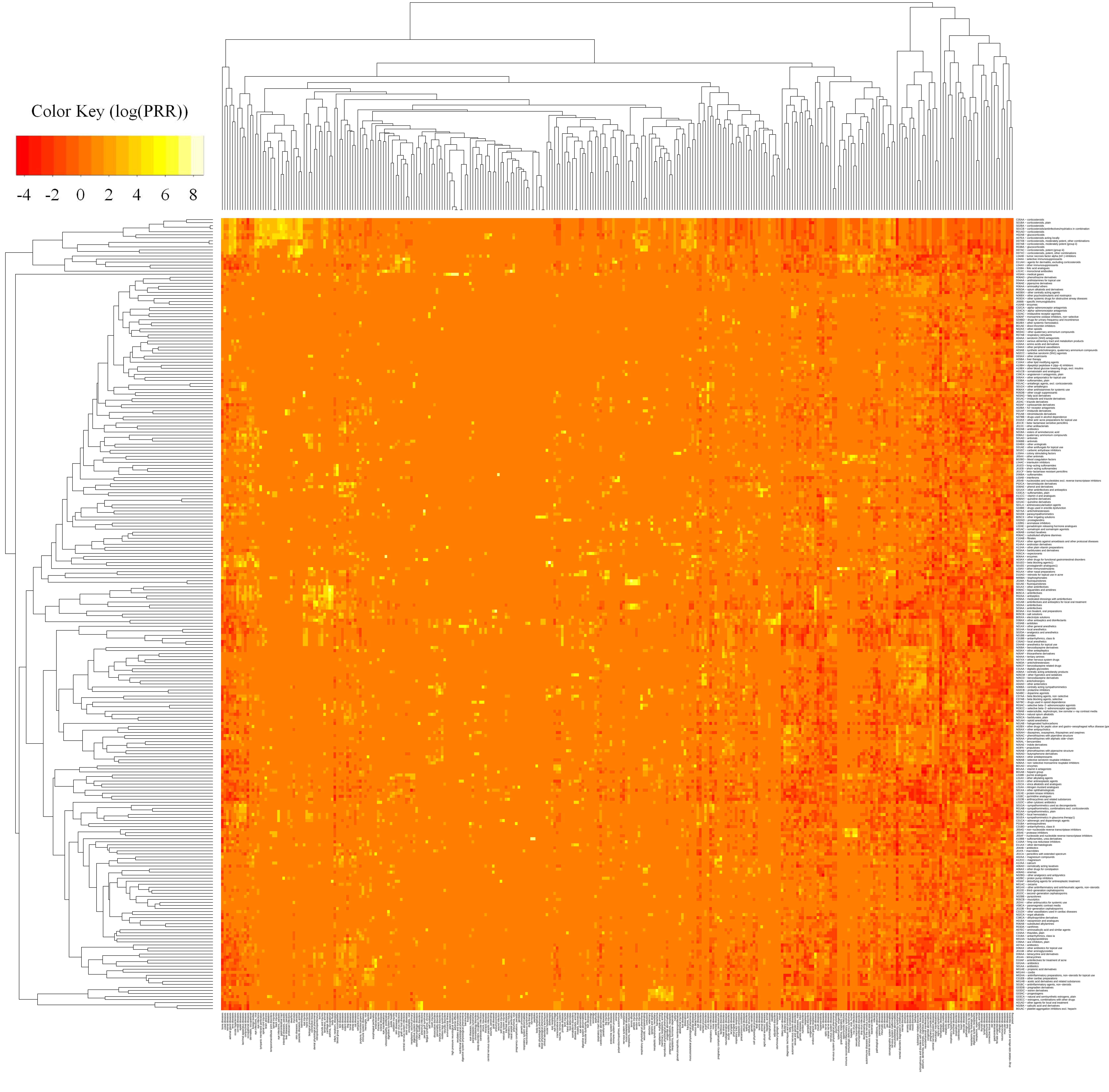

Fig. 2. Heat map of drug classes and ADE classes(based on the class signals)

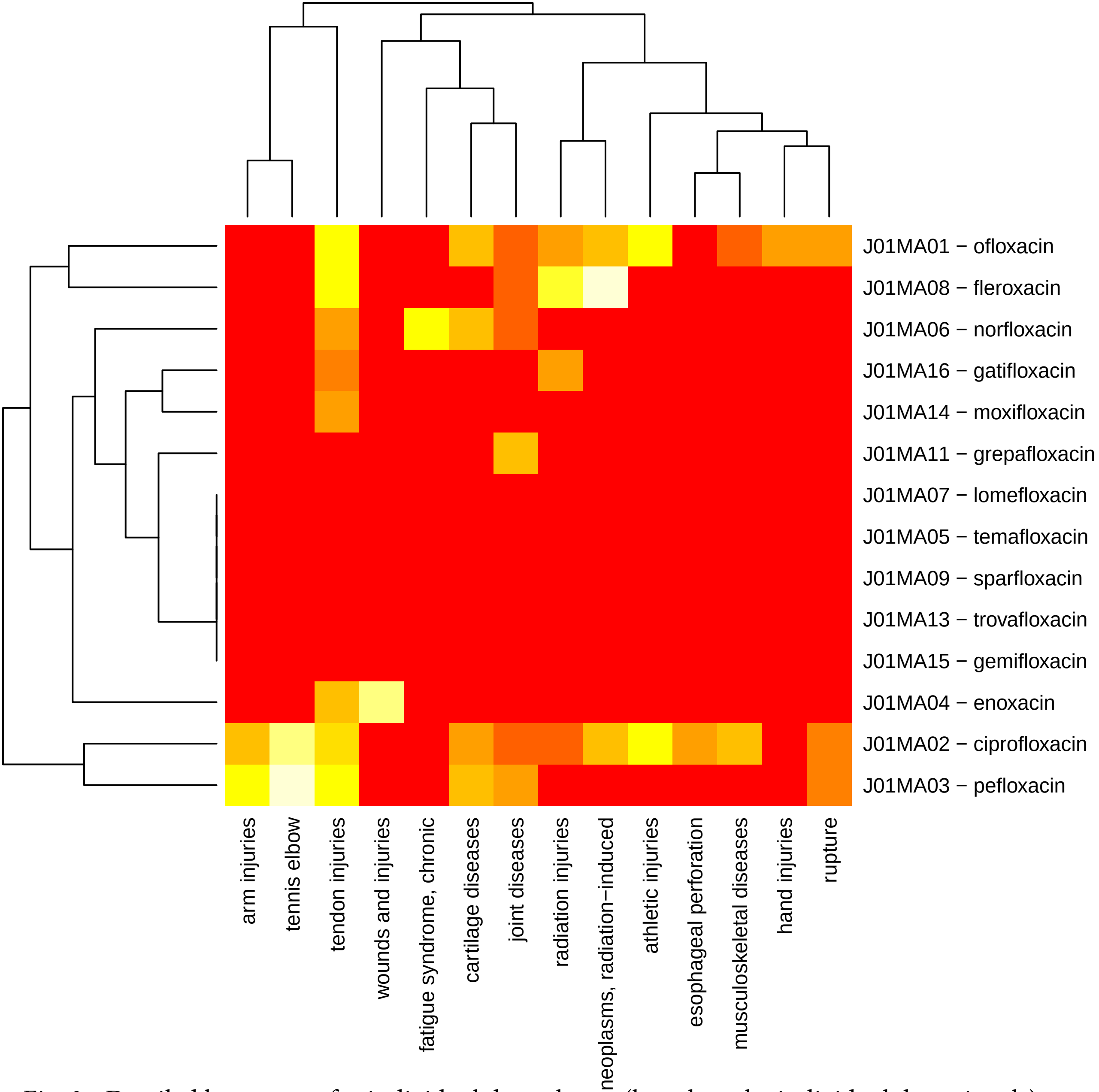

Fig. 3. Detailed heap maps for individual drug classes (based on the individual drug signals) Fluoroquinolones, ADE classes and drugs

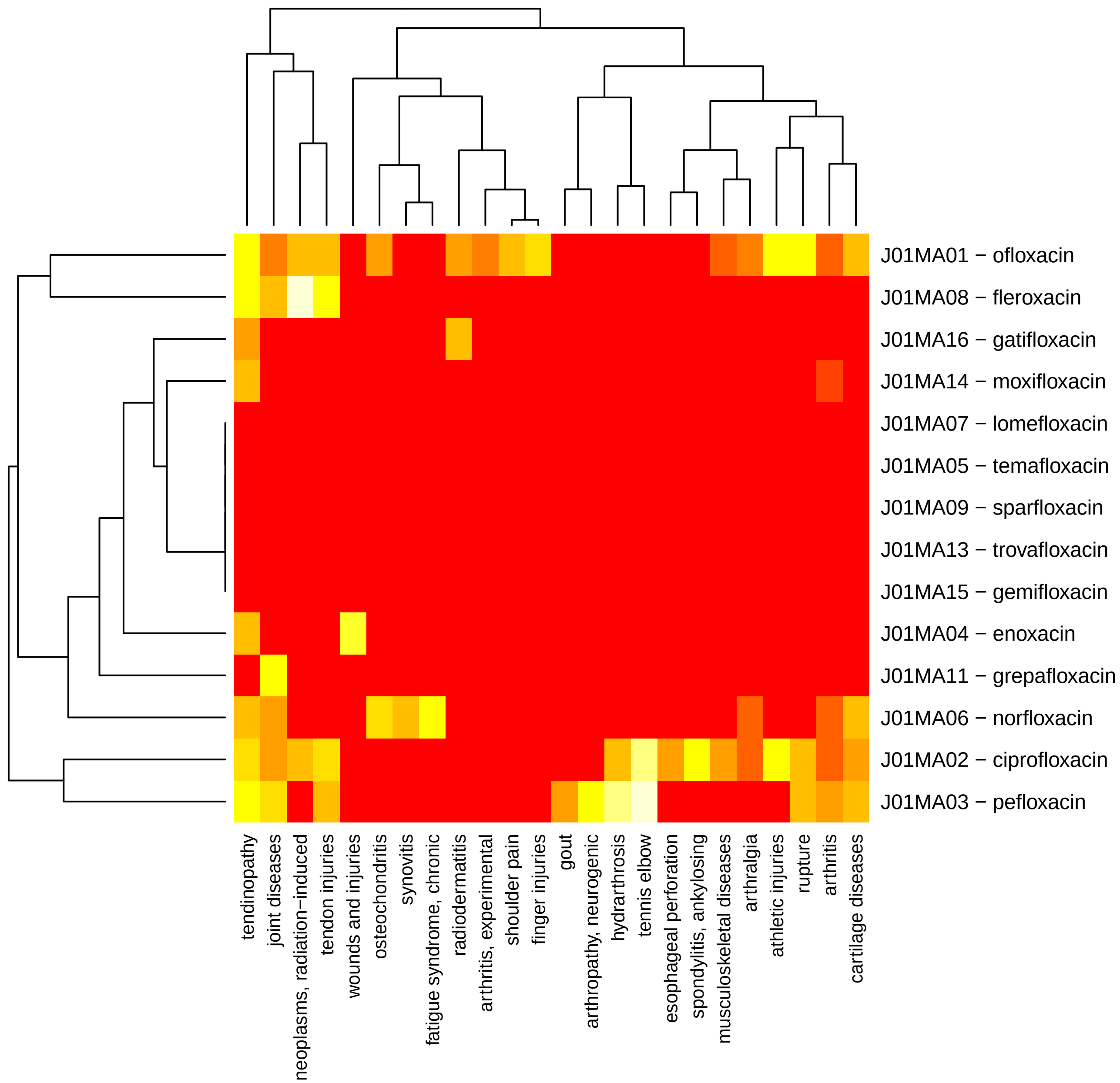

Fig. 3. Detailed heap maps for individual drug classes (based on the individual drug signals) Fluoroquinolones, ADEs and drugs

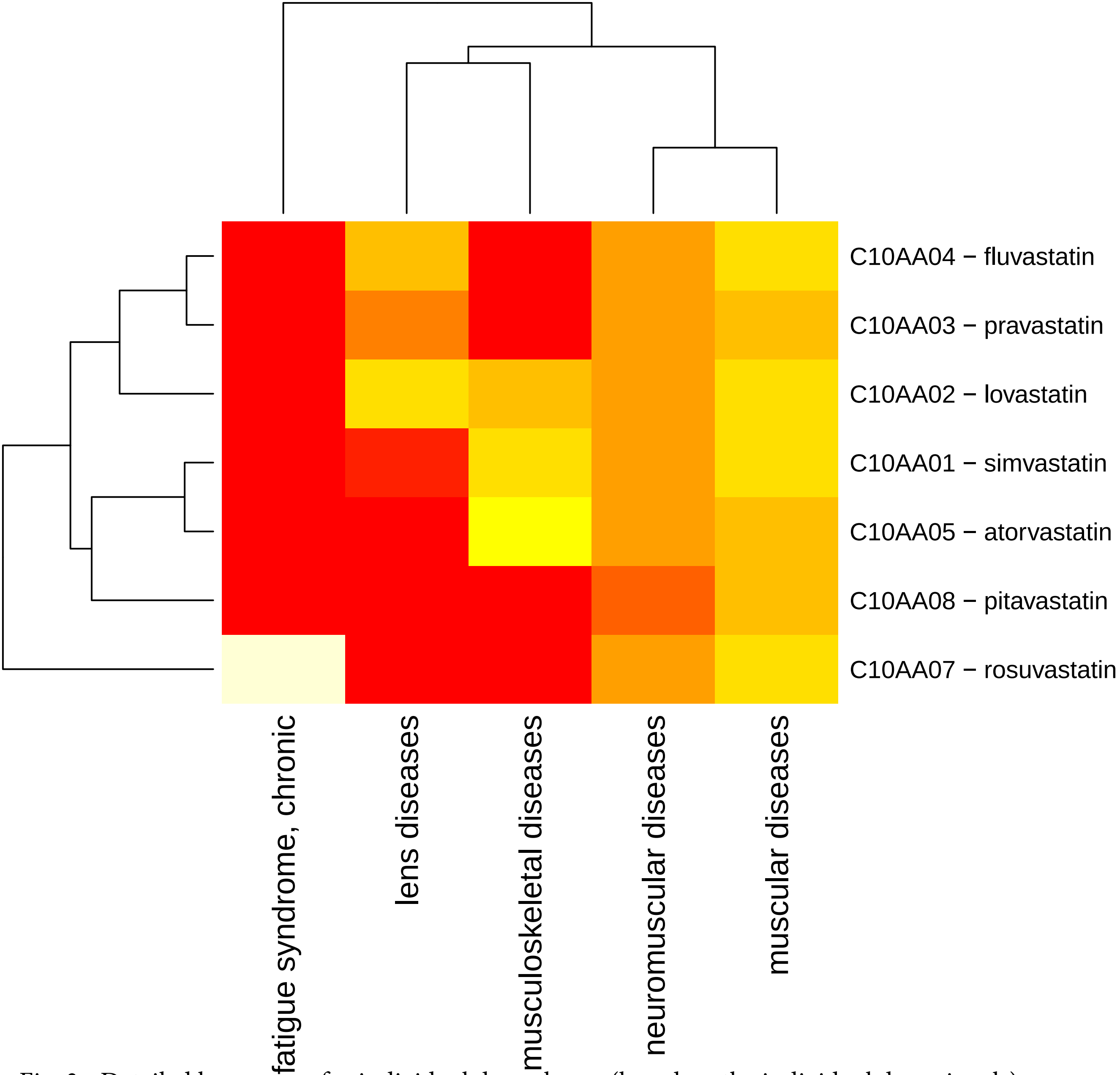

Fig. 3. Detailed heap maps for individual drug classes (based on the individual drug signals) Statins, ADE classes and drugs

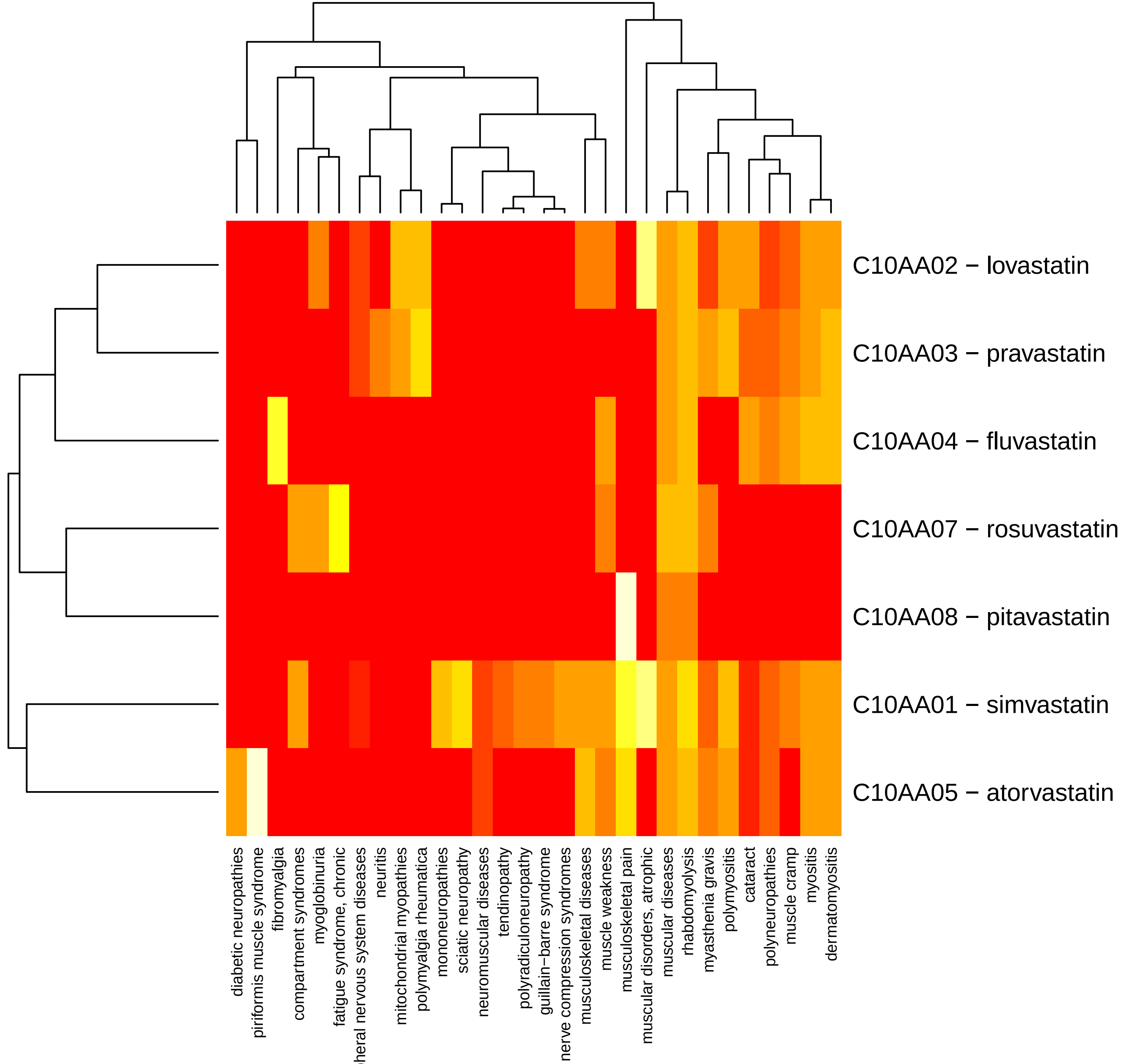

Fig. 3. Detailed heap maps for individual drug classes (based on the individual drug signals) Statins, ADEs and drugs
